# Evaluation of Physicochemical Properties of a Hydroxyapatite Polymer Nanocomposite for Use in Fused Filament Fabrication

**DOI:** 10.3390/polym15193980

**Published:** 2023-10-03

**Authors:** Ngoc Mai Nguyen, Akesh Babu Kakarla, Satya Guha Nukala, Cin Kong, Avinash Baji, Ing Kong

**Affiliations:** 1 Advanced Polymer and Composite Materials Laboratory, Department of Engineering, School of Computing, Engineering and Mathematical Sciences, La Trobe University, Bendigo, VIC 3552, Australia; 2 Department of Biomedical Sciences, University of Nottingham Malaysia Campus, Semenyih 43500, Selangor, Malaysia; 3 Department of Engineering, School of Computing, Engineering and Mathematical Sciences, La Trobe University, Melbourne, VIC 3086, Australia

**Keywords:** nanohydroxyapatite, polylactic acid, 3D printing, polymer nanocomposites, fused filament fabrication

## Abstract

Over the last decade, there has been an increasing interest in the use of bioceramics for biomedical purposes. Bioceramics, specifically those made of calcium phosphate, are commonly used in dental and orthopaedic applications. In this context, hydroxyapatite (HA) is considered a viable option for hard tissue engineering applications given its compositional similarity to bioapatite. However, owing to their poor mechanobiology and biodegradability, traditional HA-based composites have limited utilisation possibilities in bone, cartilage and dental applications. Therefore, the efficiency of nano HA (nHA) has been explored to address these limitations. nHA has shown excellent remineralising effects on initial enamel lesions and is widely used as an additive for improving existing dental materials. Furthermore, three-dimensional printing (3DP) or fused deposition modelling that can be used for creating dental and hard tissue scaffolds tailored to each patient’s specific anatomy has attracted considerable interest. However, the materials used for producing hard tissue with 3DP are still limited. Therefore, the current study aimed to develop a hybrid polymer nanocomposite composed of nHA, nanoclay (NC) and polylactic acid (PLA) that was suitable for 3DP. The nHA polymer nanocomposites were extruded into filaments and their physiochemical properties were evaluated. The results showed that the addition of nHA and NC to the PLA matrix significantly increased the water absorption and contact angle. In addition, the hardness increased from 1.04 to 1.25 times with the incorporation of nHA. In sum, the nHA-NC-reinforced PLA could be used as 3DP filaments to generate bone and dental scaffolds, and further studies are needed on the biocompatibility of this material.

## 1. Introduction

Polymer nanocomposites (PNCs) are attracting increasing attention in the fields of tissue engineering [1] and regenerative medicine [2]. PNCs can be utilised to create frameworks, forming a 3D system through which cells can grow, separate and develop into functional organisations [1]. In recent years, PNCs based on biodegradable polymers have played an essential role in material technology, the packaging industry, agriculture and the medical device industry because they exhibit excellent properties and potential for structural deformation [3].

Polylactic acid (PLA) is well acknowledged among the available biodegradable polymers owing to its decomposition process [4]. Furthermore, PLA can be decomposed into different constituents without adversely affecting the surrounding environment, using metabolic routes for deformation [4,5]. Moreover, PLA is a transparent thermoplastic aliphatic polyester with outstanding optical features, flexural strength of about 140 MPa [6], Young’s modulus around 5–10 GPa [7] and good processability (with low shrinkage, which does not cause product deformation); it is also fully biodegradable [8,9,10]. Therefore, researchers have found many promising applications of PLA, especially in biomedical applications [11,12]. Its outstanding biocompatibility ensures that no inflammatory response are elicited within the encompassing tissue or in the human body because of dismissal [13,14,15]. The human body can metabolise intermediate lactic acid, which is non-toxic and safe. However, previous studies have reported that cells cultured on PLA surfaces failed to respond to in vitro cell culture tests; therefore, PLA composites have been applied to enhance the cell-material attraction, mechanical resistance and debasement rate [16]. For instance, nanohydroxyapatite (nHA) or b-tricalcium phosphate-based PLA composites have been used to promote osteoconduction and induce bone development [17]. Excellent mechanical features were also observed when nHA and silver nanoparticles were added to a softened PLA matrix [18]. Therefore, nHA has been used as a filler to improve the bioactivity of PLA without reducing its mechanical strength.

Similarly, NCs or clay minerals have been broadly utilised to reinforce polymer matrix composites and improve the mechanical, thermal and anti-corrosive features. An addition of NC at low loading is also an excellent way to enhance the mechanical strength [19,20,21], barrier strength [22,23] and thermal features of PLA polymers [19,24,25]. Researchers have found that using NC at different percentages, ranging from 1% to 6%, could help increase the mechanical and optical properties of PLA and improve the performance of PLA in barrier tests for industrial production and packing applications [26].

HA [Ca_10_(PO_4_)_6_(OH)_2_] is a calcium phosphate that has a morphology and composition similar to that of human hard tissues [27]. HA also has the same chemical and mechanical properties as bones, teeth, dentine, enamel and calcified parts of tendons [28]. Typically, HA has a hexagonal structure and a stoichiometric Ca/P ratio of 1.67, such as that of bone apatite [29,30,31]. HA is an inorganic mineral with a carbonated apatite structure present in human bone and teeth with a minority of deficient calcium [32]. Moreover, calcium HA is insoluble in environments with acid–base balance, i.e., pH 7.4, which is the average pH of the human body for various biological processes, especially blood oxygenation. However, it dissolves insignificantly at a pH of <6.5 [33]. The advantage of nHA is that it can be synthesised into various forms. Different forms of synthesised nHA, such as dense ceramic [34], powder [35], coating material [36] or porous material [37], are suitable for diverse applications. Therefore, nHA is the most commonly applied bioceramic in the field of prosthetics [8] and as a delivery carrier for proteins [38], peptides [39], genes [40] and drugs [41], owing to its excellent properties and flexible structure. Additionally, nHA is a promising bioceramic for bone tissue engineering [42], dental applications [43] and stem cell differentiation [44].

Turk et al. [45] demonstrated that nHA produced using the sonochemical method displayed hexagonal and rod-like nanostructures. Similarly, Huang et al. [46] reported the generation of spherical-shaped nHA through a single-step hydrothermal process. In another study by Xing et al. [47], rod-shaped nHA was obtained through ultrasonication, and fibrous nHA through precipitation. However, the shape of nHA is a vital specification for biomedical applications. Hence, it is necessary to choose the appropriate synthesis method to produce a viable shape suitable for end applications [48]. In another report, the thermal properties of nHA synthesised from eggshells were investigated using thermogravimetric analysis (TGA) [49]. The weight loss curve had two different decomposition stages: about 3% weight loss at 0–200 °C was attributed to residual water loss, and thermal decomposition of nHA occurred at around 212–250 °C, resulting in weight loss of about 8%. Consequently, a total decomposition of 12% was detected at a maximum temperature of 600 °C [49]. Webster et al. [50] reported that small nHA particles (67 nm) essentially improved osteoblast attachment, as opposed to nHA particles measuring >100 nm after culturing for 4 h, while suppressing fibroblast bonds. The nanophase ceramics also demonstrated the highest adsorption of vitronectin, a protein promoting osteoblast attachment. Additionally, given its high bioactivity and biocompatibility, nHA is applicable to implantation in tissue engineering [51].

In dentistry, nHA has been used as a dental implant coating material because it can inhibit the growth of Gram-positive and Gram-negative bacteria [52]. Furthermore, in an inflammation study, nHA played an essential role as a modulator of monocytes and macrophages responsible for early-stage inflammatory reactions [53]. It was found that the advantage of implants with a thin coating of nHA could be attributed to a reduction in the inflammatory response [53]. In another study, a gel product with a 30–50% concentration of hydrogen peroxide, enriched with fluoride calcium and nHA, was applied as a bleaching agent [54]. This gel prevented hypersensitivity after bleaching [55]. Moreover, nHA paste could be used to repair microscopic surface imperfections and subsurface pores in the enamel that allow bleaching agents to penetrate and thus cause sensitivity [56].

In 3D printing, filaments are vital to the production of 3D models [57]. Filaments are fed through the extruder head via rollers and gears; as they melt, they are deposited on the build platform [57]. Therefore, 3D printers need solid and durable filaments that can yield an excellent final product with adequate physical properties. Materials such as PLA and acrylonitrile butadiene styrene (ABS) are generally used as 3D printing filaments. Furthermore, advanced materials for additive manufacturing such as polycaprolactone (PCL) [58], nylon [59], carbon fibre [59], glass fibre [60] and many others [60,61] are available in the market. However, most of these materials are more efficient in producing the products for real world applications rather than for prototyping. Therefore, researchers and manufacturing industries have been investigating various nanocomposite-based filaments to design and enhance the physical properties of 3D models that can be used as original equipment manufacturers in the automobile and biomedical industries.

Miron et al. [62] examined the properties of PLA filament produced using a Filabot EX2 extruder. The extruded filaments had an average diameter of 2.0 mm, and displayed good mechanical and biodegradable properties. Bhagia et al. [63] reported the tensile properties of wood-reinforced PLA filaments for 3D printing applications. The Filabot EX2 single-screw extruder specifications used were 1.58 screw diameter, 1.27 pitch length/diameter ratio of 12 and compression ratio of 2.5:1. The filaments thus manufactured had dimensions of 2.6 ± 0.03 mm. Further, the measurement of the dimensions was confirmed using a micrometer. The filaments were used to produce 3D printed samples with a tensile strength of 50 MPa. Chang et al. [64] described the fabrication of PCL–HA composite filaments for the printing of bone scaffolds. The filaments were produced using a Filabot EX2 extruder with a nozzle diameter of 1.75 mm. The composites were extruded into filaments at 100 °C. The produced filaments were successfully used to design 3D models with varied concentrations of HA polymer composites. However, most of the studies have examined HA-PLA scaffolds fabricated using various technologies such as electrospinning, freeze drying and solvent casting. There is still a dearth of research on polymer composites based on 3D printing. Therefore, in the present study, hybrid nHA-NC-reinforced PLA composite filaments were used to produce a porous scaffold via 3D printing. The composition and structures of the printed nHA-NC-PLA filaments were systematically analysed to determine their printability and chemical and mechanical properties.

## 2. Materials and Methods

### 2.1. Materials

Calcium hydroxide (Ca(OH)_2_, analytical reagent ((AR), ≥95%), phosphoric acid (H_3_PO_4_, AR, ≥85 wt% in H_2_O) with a relative density of 1.685 g·cm^−3^, NC (Nanomer 1.31 PS, montmorillonite clay containing 0.5–5 wt% 3-aminopropyltriethoxysilane and 15–35 wt% octadecyl amine), ammonia solution (NH_4_OH, AR) and chloroform (AR, 99%) were purchased from Sigma Aldrich (Melbourne, Australia). PLA feedstock material was purchased from 3D Nielsen (Helsingør, Denmark).

### 2.2. Synthesis of Nanohydroxyapatite (nHA) Particles

**Figure 1:**
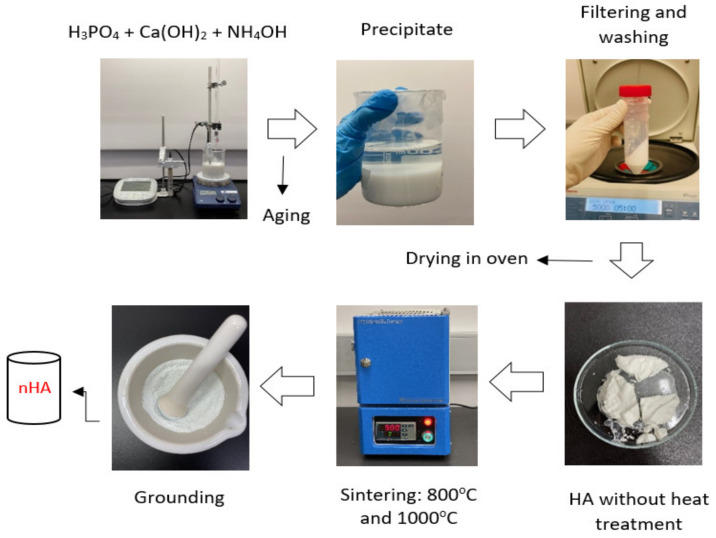
Synthesis of nHA particles using the wet chemical precipitation method.

**Figure 2:**
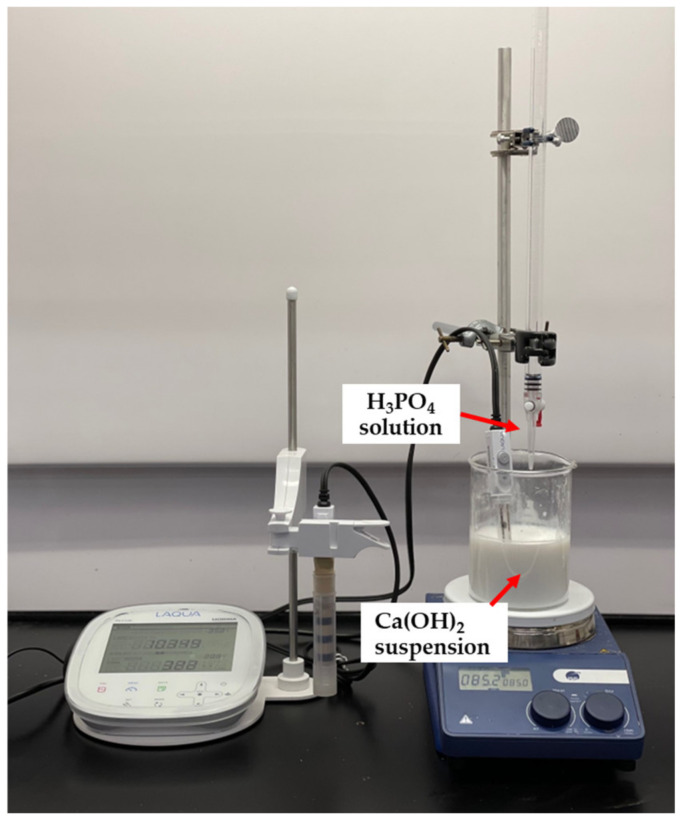
Adding H_3_PO_4_ solution dropwise to Ca(OH)_2_ suspension.

The synthesis of nHA particles was based on the wet chemical precipitation method using Ca(OH)_2_ and H_3_PO_4_ as starting materials [65]; the procedure is illustrated in Figure 1. To ensure the stoichiometric molar ratio of nHA was 1.667, the concentrations of calcium and phosphorus were maintained at 1.0 M and 0.6 M, respectively. NH_4_OH solution was applied to adjust the pH of the mixing solution to around 10.0 to prevent the formation of a calcium-deficient HA structure [65]. Firstly, to prepare a 1.0 M Ca(OH)_2_ suspension, 15.6 g of Ca(OH)_2_ powder was added to 200 mL DI water in a glass beaker, followed by stirring with a magnetic stirrer at 35 °C, 300 rpm for 1 h. A 0.6 M H_3_PO_4_ solution was prepared by adding 8.21 mL of H_3_PO_4_ to 200 mL of DI water in another glass beaker with manual stirring at room temperature for 5 min. Then, the H_3_PO_4_ solution was added dropwise to the Ca (OH)_2_ suspension at a rate of 5.5 mL·min^−1^ with continuous stirring by a magnetic stirrer and pH monitoring using a pH meter. This procedure is demonstrated in Figure 2. Once the H_3_PO_4_ solution was added entirely to the Ca(OH)_2_ suspension, the NH_4_OH solution was added dropwise to the mixing solution to maintain the pH at about 10. A white precipitate began to form. The mixing solution was continuously stirred for 2 h at 85 °C, 850 rpm and covered to prevent any potential splashing or contamination from the surroundings. The beaker containing the mixing solution was left in the fume hood for 3 days for maturation.

Next, the precipitate was filtered and washed three times with DI water using a centrifuge. The acquired precipitate was stored in a Petri dish and dried in the oven overnight at 60 °C to obtain semi-dry precipitates. The semi-dry precipitates were divided into two parts in different ceramic bowls for sintering in an electric furnace (CF1100 Muffle Furnace, Across International, Livingston, NJ, USA) at 800 °C and 1000 °C, respectively for 2 h. After sintering, the products were allowed to cool in the furnace for 1 h. Finally, the products were ground to powder form using mortar and pestle. The synthesised nHA powders were labelled according to the thermal conditions they were treated with, as listed in Table 1.

### 2.3. Preparation of nHA-NC-PLA Nanocomposite Filament

The PLA nanocomposites were prepared using the solvent casting method in which chloroform was used as the solvent [66]. NHA1000 synthesised in the previous experiment, and NC was chosen for the nanoparticles to produce PLA nanocomposites. The PLA nanocomposites with different compositions are shown in Table 2.

First, PLA pellets, according to the wt% presented in Table 2, were dissolved in chloroform for 24 h in different beakers. Once the PLA pellets were dissolved entirely, nHA and NC were added with ultrasonication for 10 min. Further, the homogenous solutions were cast in different Petri dishes and oven-dried at 40 °C for 72 h to evaporate the solvent. Finally, the cast films were washed with DI water and diluted ethanol to remove any residues.

### 2.4. 3D Printing of Produced Filament

**Figure 3:**
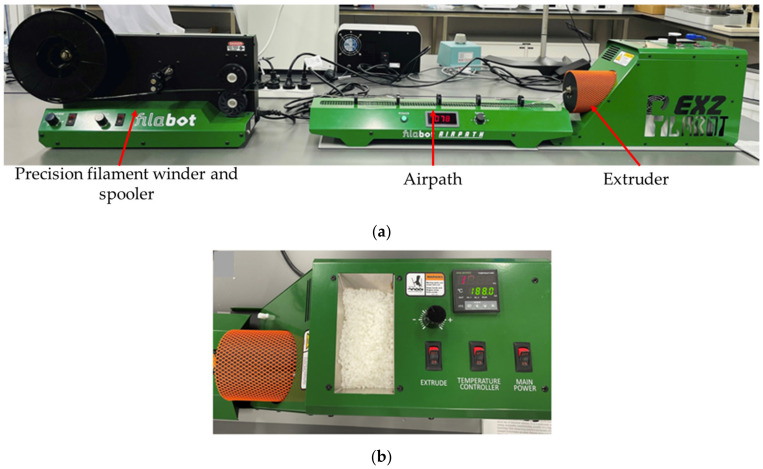
Producing the neat PLA and PLA nanocomposite filaments by using the (**a**) Filabot system with (**b**) EX2 filament extruder.

**Figure 4:**
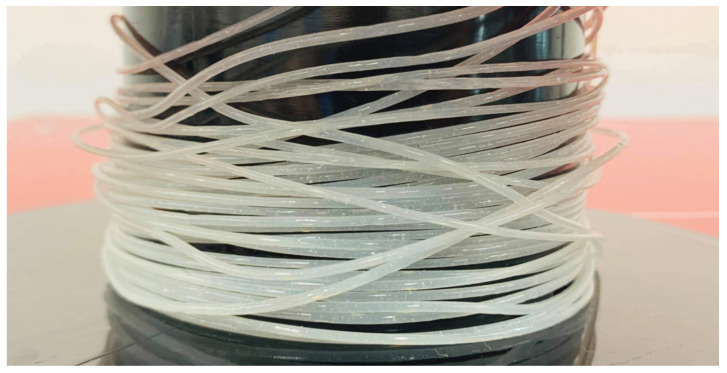
PLA nanocomposite filament spool.

The nanocomposite filaments were prepared by the melt extrusion procedure with the Filabot system (Filabot, Barre, VT, USA), as illustrated in Figure 3a,b, including the Filabot EX2 filament extruder with the support of Filabot Airpath and Filabot Spooler—Precision Filament Winder. First, PLA nanocomposite films were cut into 1 mm to 2 mm sizes with mechanical scissors. The films were fed into the material input and melted using the extruder temperature controller. The temperature was set around 175 °C, and the diameter of the nozzle was 2.85 mm. The airpath cooled the extruded filament and it was rolled by the spooler, as shown in Figure 4, and extrusion parameters are shown in Table 3. A similar procedure was followed to extrude the neat PLA pellets at an extrusion temperature of 174 ± 1.5 °C.

### 2.5. 3D Printing of Scaffold Models

**Figure 5:**
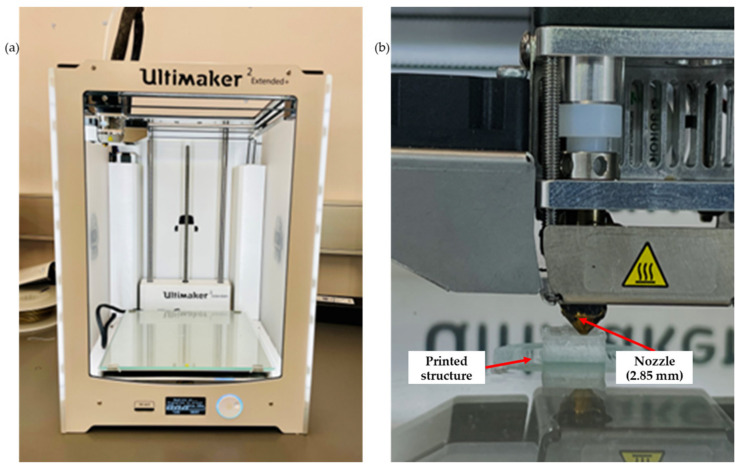
(**a**) Ultimaker 2 Extended Plus 3D printer, and (**b**) 3D printing of the scaffold model.

Three filaments, including neat PLA, PLA-nHA1-NC0.5 and PLA-nHA2, were chosen for the 3D printing. The filaments were printed using a 3D printer (Figure 5a, Ultimaker 2 Extended Plus, Ultimaker, Utrecht, The Netherlands) with fused filament fabrication (FFF) technology. The printing parameters in Table 3. The 3D printing structure of 10 × 10 × 5 mm^3^ was designed in SolidWorks (Dassault Systèmes, Waltham, MA, USA). Further, the designed structure was converted into a G-code file using a 0.17 mm single layer height and 2.0 mm pore size, respectively. The printing was conducted at room temperature. Scaffold models were acquired by printing them in a layer-by-layer format through the filaments to obtain 3D structures on the 3D printer platform, as shown in Figure 5b.

### 2.6. Characterisation

#### 2.6.1. Morphology

The morphology of different synthesised nHA powders and the fractured surface of PLA nanocomposite filaments were observed with the high-resolution Field-Emission Scanning Electron Microscope (FE-SEM) SU700 (Hitachi, Tokyo, Japan). The neat PLA and different nanocomposite filaments were manually broken into tiny pieces. The cross-section of the specimens could be acquired from fractured surfaces. The samples were attached by mounting tape and sputter coated with gold before scanning to avoid electrostatic charges under an accelerating voltage of 5.0 kV (for nHA powder) or 10.0 kV (for PLA nanocomposite filament samples) and high vacuum mode for 30 s. Additionally, the size of nanoparticles was estimated from SEM images by Fiji image processing software (ImageJ, V1.5, GNU General Public License, U. S. National Institutes of Health, Bethesda, MD, USA).

#### 2.6.2. Fourier Transform Infrared (FTIR) Spectroscopy

The chemical compositions of nHA powder synthesised with different heat treatment conditions were examined with the Cary 630 FTIR spectrometer (Agilent, Santa Clara, CA, USA). The spectra were logged in transmittance mode with a resolution of 4 cm^−1^ ranging from 500 to 4000 cm^−1^.

#### 2.6.3. Thermogravimetric Analysis (TGA)

Analyses of the thermal stability of the neat PLA and different PLA nanocomposite filaments were carried out with the Thermogravimetric Analyzer (TGA 4000, PerkinElmer, Waltham, MA, USA). The tests were conducted under a nitrogen atmosphere at a flow rate of 20 mL·min^−1^. About 3.0 mg of each sample was prepared and placed in an open alumina crucible. Then, specimens were heated from 30 to 850 °C at 10 °C·min^−1^, holding for 1 min at 30 °C and 850 °C.

#### 2.6.4. Differential Scanning Calorimetry (DSC)

The thermal properties of neat PLA and different PLA nanocomposite filaments were measured with a Differential Scanning Colorimeter (DSC 6000, PerkinElmer, Waltham, MA, USA). The samples of approximately 3.0 mg were pressed in an aluminium crucible and heated in two heating cycles under a nitrogen gas atmosphere. The first heating cycle was carried out from 30 to 200 °C at 40 °C·min^−1^, then kept for 2 min at 200 °C to erase the previous thermal history. They were then cooled from 200 to 30 °C at 20 °C·min^−1^ and held for 2 min at 30 °C to obtain the possible crystallisation processes. Furthermore, a second heating was carried out from 30 to 200 °C at 20 °C·min^−1^ and held for 2 min at 200 °C to investigate any thermogram differences from heating cycle 1.

#### 2.6.5. Compression Testing

Samples with 10 (L) × 10 (W) × 5 (T) mm^3^ dimensions (ASTM D695) were used for compression testing [67,68]. The testing was conducted using an Instron 5980 UTM (Instron, Norwood, MA, USA) furnished with a 50 kN load cell. At first, the upper and lower surfaces of 3D models were attached to the machine to ensure that slippage did not occur during testing. The stiffness of the scaffolds was measured as the slope of the initial portion of the stress–strain curve.

#### 2.6.6. Tensile Properties

The extruded filament of each composition was moulded in dumbbell shapes (ASTM D638 Type IV) with a dimension of 10 × 15 mm^2^ as per the following reference: [69]. Then, the tensile properties of the neat PLA and PLA nanocomposite filament samples were examined using the Instron 5980 Universal Testing Machine (UTM) (Instron, Norwood, MA, USA) equipped with a 50 kN load cell at a crosshead speed of 1 mm·min^−1^ [70,71].

#### 2.6.7. Hardness

All samples for the hardness examination were produced from different filaments using the PCH-600D hydraulic lamination hot press (Henan Chuanghe Laboratory, Henan, China) with a dual temperature controller at 180 °C for 5 min at 5 MPa pressure. These specimens were cut into a rectangular shape with dimensions of 15 (L) × 5 (W) × 1 (T) mm^3^. Then, the surface hardness measurement was conducted using the Scan-20 G5 (Struers, Copenhagen, Denmark) hardness tester for the neat PLA and various PLA nanocomposite samples, to which a load of 100 g/force was applied for 15 s. Microhardness measurements were taken from the top surface to the interface and from the interface to the depth of 2.5 mm within the substrate. The results acquired from the equipment were in the Vickers hardness (HV) unit. Then, they were converted to a GPa unit by multiplying by 0.0098 [72].

#### 2.6.8. Water Absorption

The water absorption experiment followed the ASTM D570 standard [69]. The neat PLA and PLA nanocomposite filaments were cut into small pieces, initially weighed and immersed in DI water at ambient conditions. These specimens were periodically weighed at various intervals (2, 4, 6 and 8 days) to determine the water uptake by sample. The percentage of absorbed water (*W*) in the neat PLA and PLA nanocomposite filament specimens was calculated using Equation (1):

(1) $$W(\%)=[(W_{1}-W_{0})/W_{0}]\times 100$$

where $W_{1}$ and $W_{0}$ are the sample weights after and before being immersed in the water.

#### 2.6.9. Water Contact Angle

The wettability of the neat PLA and various PLA nanocomposite filaments was evaluated by measuring the water contact angle using the sessile drop technique. At first, a droplet was placed on the sample surface using a micrometre syringe. Next, the water contact angle was determined by scanning the droplet profile for 20 s using the Theta Flex optical tensiometer (Biolin Scientific, Frölunda, Sweden). Maintaining the size of the water droplet at about 2–2.5 µL was essential to avoid the effects of weight.

#### 2.6.10. Printability

**Figure 6:**
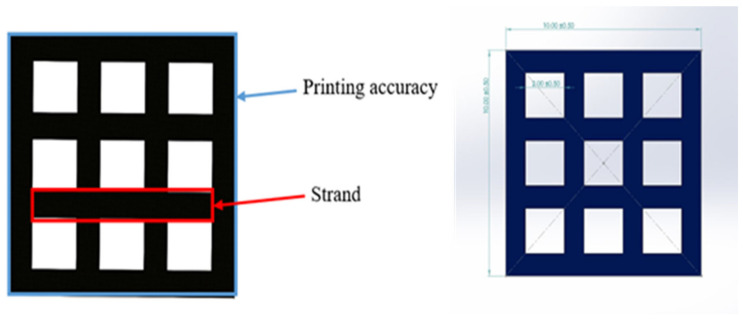
3D scaffold model designed in SolidWorks.

To access the printability properties, the 3D printed scaffold models were captured using a 24-megapixel (MP) camera (Canon, EOS 200D II, Tokyo, Japan). The strand printability and printing accuracy, as described in Figure 6, were estimated from the acquired images of different scaffold models using Fiji image processing software (ImageJ, V1.5, GNU General Public License, USA).

The strand printability was defined to examine how uniformly the printed strands contrasted with the designed strand. This parameter was calculated utilising Equation (2) [67]:

(2) $$Strand\;printability=\frac{length\;of\;printed\;strand\;after\;cooling\;to\;room\;temperature}{length\;of\;designed\;strand}$$

The printing accuracy of each scaffold model was measured following Equation (3) [67]:

(3) $$Printing\;accuracy\;\left(\%\right)=\left(1-\frac{\left|A_{i}-A_{o}\right|}{A_{o}}\right)\times 100$$

where $A_{i}$ is the initial area of the designed model, and $A_{o}$ is the overall area of the 3D printed model.

## 3. Results and Discussion

### 3.1. Morphology

**Figure 7:**
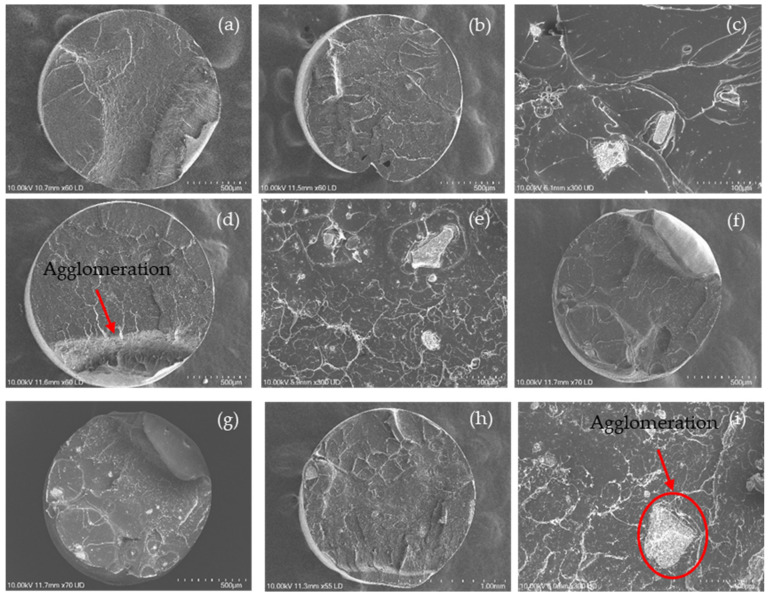
SEM images of the fractured surface of (**a**) PLA; and PLA nanocomposite filaments including (**b**,**c**) PLA-nHA1, (**d**,**e**) PLA-nHA1-NC0.5, (**f**,**g**) PLA-nHA2 and (**h**,**i**) PLA-nHA2-NC0.5.

The dispersion of the nanoparticles in the PLA nanocomposite filaments was observed by SEM, as shown in Figure 7. Regarding the neat PLA filament (Figure 7a), the cross-section of the filament was visualised as a smooth fractured surface with slight roughness and typical fracture properties of a brittle material [73].

However, other SEM images from Figure 7b–i provide information regarding the distribution of nanoparticles on the cross-section surface of the nanocomposite filament. Nanoparticles with irregular shapes and various sizes were observed on the surface structure of fractured nanocomposite filaments with an uneven dispersion. These nanoparticles led to roughness on the surface of the fractured nanocomposite filaments compared with that on the surface of the neat PLA filaments. Furthermore, the concentration of nanoparticles on the fractured surface became more evident as the wt% of the nanoparticle (nHA and NC) content increased. It was evident that the fractured surface of the filaments containing NC (PLA-nHA1-NC0.5 and PLA-nHA2-NC0.5) was smoother than that of the filaments without NC (PLA-nHA1 and PLA-nHA2). 

Further, there were two dark spots on the cross-section surface of the PLA-nHA1 filament, as illustrated in Figure 7b, which were the air gaps on the filaments produced by the extrusion procedure. The air gaps on the surface may have been generated by the unstable loading of PLA-nHA1 nanocomposite during the extrusion process. 

The agglomeration of nanoparticles in nanocomposite filaments is shown in Figure 7c,e,g,i. The SEM images show that the nanoparticles were distributed on the PLA nanocomposite filaments in both isolated and agglomerated forms, leading to poor interfacial adhesion between the nanoparticles and PLA. In fact, Zare et al. [74] found that the agglomeration or aggregation of nanoparticles significantly reduced the interfacial or interphase and tensile characteristics of nanocomposites by reducing the specific surface area and effective volume fraction of the nanoparticles.

### 3.2. FTIR

**Figure 8:**
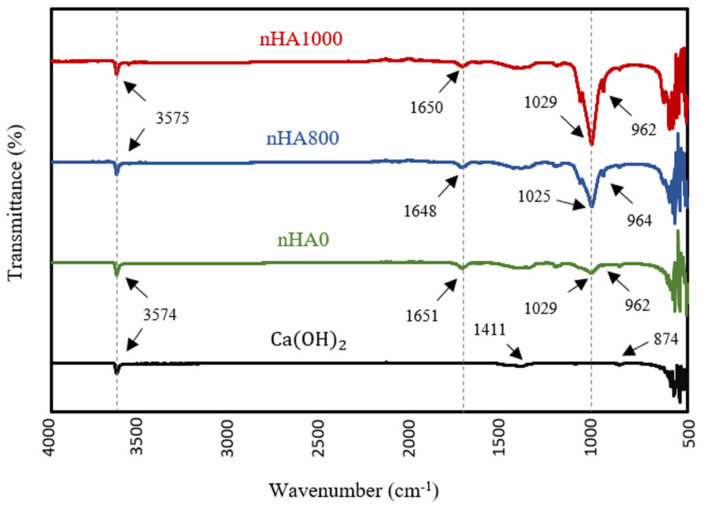
FTIR spectra of Ca(OH)_2_ and nHA synthesised at different heat treatment conditions.

FTIR analysis was performed to identify the chemical functional groups of various synthesised powders and to confirm the production of nHA powder. The FTIR specta, shown in Figure 8, confirm the formation of nHA produced using the wet chemical precipitation method at different heat treatment conditions, including semi-dry nHA or nHA with no heat treatment (nHA0), nHA calcined at 800 °C (nHA800) and nHA calcined at 1000 °C (nHA1000). The variations in the spectra between Ca(OH)_2_ and nHA were obvious. However, there was no significant difference in the spectra of nHA0, nHA800 and nHA1000. The clear peaks in the regions from 3574 cm^−1^ to 3575 cm^−1^ originated from O-H stretching [75]. These peaks confirmed the presence of the ${OH}^{-}$ group in the synthesised nHA powder and Ca(OH)_2_. Further, ${PO}_{4}^{3-}$ groups were well defined with sharp peaks in the region of 1025 cm^−1^ or 1029 cm^−1^ and at around 960 cm^−1^, in line with the results of Destainville et al. [76] and Raynaud et al. [77]. The peaks of the ${PO}_{4}^{3-}$ group could not be seen in the pattern of Ca(OH)_2_.

The transmittance intensity of the ${PO}_{4}^{3-}$ group peak increased as the calcination temperature increased. However, the peaks of the ${OH}^{-}$ and ${CO}_{3}^{2-}$ groups indicated the presence of similar transmittance intensities between the different synthesised HA powders. Additionally, in the FTIR patterns of nHA0, nHA800 and nHA1000, the ${CO}_{3}^{2-}$ group showed weak bands at around 1650 cm^−1^ [77], further confirming the presence of ${CO}_{3}^{2-}$ ions in the molecular structure of nHA. Conversely, the spectrum of Ca(OH)_2_ contained two weak peaks at 1411 and 874 cm^−1^, validating the two vibration modes of C−O originating from the ${CO}_{3}^{2-}$ group [78,79]. Therefore, the chemical properties of the nHA and Ca(OH)_2_ were clearly distinguished by FTIR.

### 3.3. TGA

**Figure 9:**
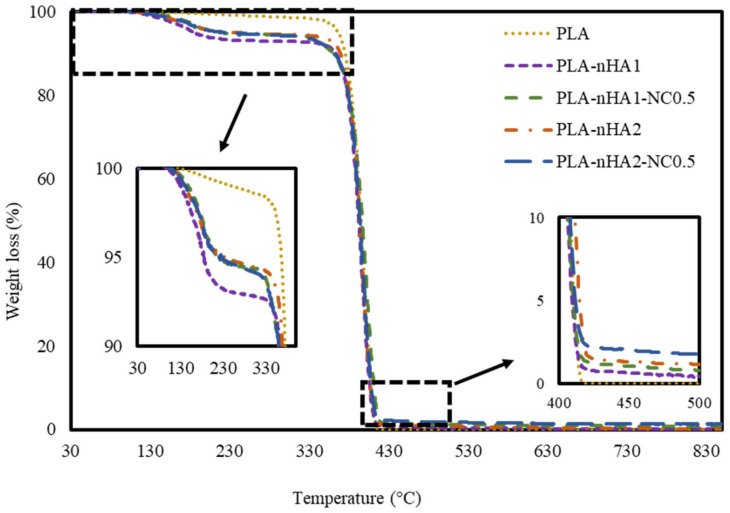
TGA curves of the neat PLA and PLA nanocomposite filaments.

The thermal degradation of the neat and PLA nanocomposite filaments was examined at a temperature range of 30–850 °C. Figure 9 shows the TGA curves of the PLA and PLA nanocomposite filaments. The thermal decomposition of filament samples involved two stages: from 30 to 350 °C and from 350 to 850 °C. In the first stage (30–350 °C), the initial weight loss in PLA samples was less than that in the nanocomposite samples by 2.3%. This result can be explained by the vaporisation of residual water acquired from the synthesis processes of different nanocomposites. Regarding the PLA nanocomposite filament samples, the PLA-nHA1 sample lost around 8.2%, whereas the PLA-nHA1-NC0.5, PLA-nHA2 and PLA-nHA2-NC0.5 samples lost around 6.5% in weight at the temperature range of 30 to 350 °C. These results confirm that the PLA-nHA1-NC0.5, PLA-nHA2 and PLA-nHA2-NC0.5 samples had higher decomposition temperatures than the PLA-nHA1 sample. The higher decomposition rate in the initial stages occurred due to the breakdown of the hydroxyl groups in the fillers as well as the matrix [80]. In the second stage, the thermal decomposition of all samples occurred with more than 98% weight loss, mainly contributed by the thermal degradation of the PLA polymer and NC. The PLA sample completely decomposed at 416 °C, as shown in Figure 9. In a study reported by Kumar et al. [81], NC lost 30% of its weight at a temperature range of 30 to 800 °C. Further, the remaining components in the nanocomposite samples were nHA particles (PLA-nHA1, PLA-nHA2) and a small amount of NC (PLA-nHA1-NC0.5, PLA-nHA2-NC0.5). Finally, the residual weight percentages of PLA-nHA1, PLA-nHA1-NC0.5, PLA-nHA2 and PLA-nHA2-NC0.5 at 850 °C were 0.2, 0.8, 1.0 and 1.3%, respectively.

### 3.4. DSC

**Figure 10:**
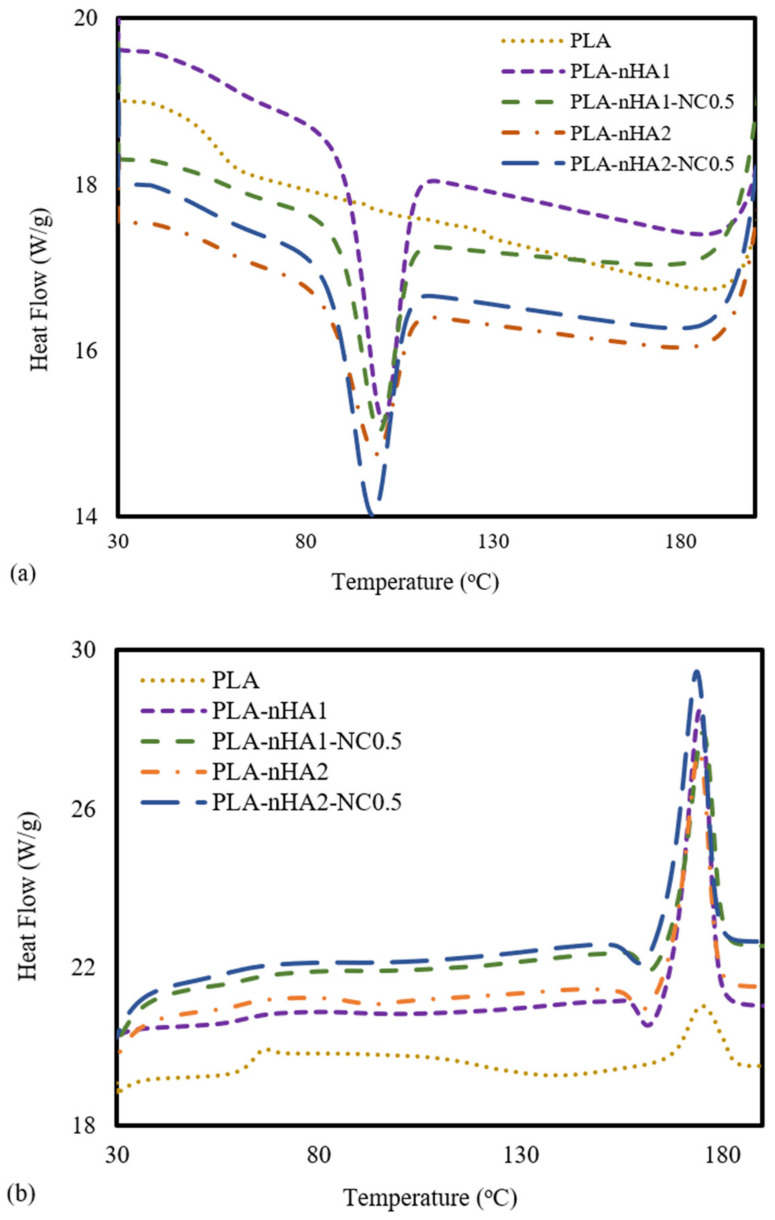
DSC analysis at the (**a**) cooling stage and (**b**) second heating stage of the neat PLA and PLA nanocomposite filaments.

DSC analysis was carried out to determine the thermal behaviours of the neat PLA and PLA nanocomposite filaments. The DSC data from the cooling and the second heating processes are shown in Figure 10a,b. For PLA, no peak in the DSC cooling curve was observed (Figure 10a), meaning the crystallisation of the PLA sample did not occur during the cooling process. Furthermore, the PLA nanocomposites reinforced with nanoparticles (nHA and NC) showed peaks at around 100 °C, and the crystallisation temperature (T_c_) of a PLA nanocomposite sample increased when the weight percentage of the nanoparticles in the PLA polymer decreased [82]. The T_c_ values of the PLA-nHA1, PLA-nHA1-NC0.5, PLA-nHA2 and PLA-nHA2-NC0.5 nanocomposites were 103, 102, 101 and 96 °C, respectively. Moreover, the glass transition of PLA occurred at around 55 °C (T_g_), while there was no evidence of glass transition on the cooling curves of the composites [66,83].

Regarding the subsequent heating data of the PLA and PLA nanocomposite filaments shown in Figure 10b, there was no significant difference in their melting points, and their melting temperatures (T_m_) were close to 174 °C. However, PLA-nHA2-NC0.5 had a higher heat flow at 29 W·g^−1^, resulting in rapid melting. Nanocomposites with high heat flow tend to melt quickly. However, the neat PLA had a low heat flow at 20 W·g^−1^. Consequently, the nanoparticles did not have a substantial effect on the melting temperature of the PLA polymer. The peaks of T_g_, T_c_ and T_m_ are listed in Table 4. Furthermore, a weak peak at 68 °C in the second heating curve of PLA was observed, indicting the transition to glass. Nevertheless, the transition did not occur in the second heating process for any of the PLA nanocomposites. According to Krishnamachari et al. [84] and Zheng et al. [85], the intermolecular interaction, chain flexibility and nHA filler interaction with the matrix influence the T_g_. Therefore, the glass transition of fillers incorporated with a matrix limited the decomposition. Overall, since nHA aids the formation of rigid phases that regulate shape memory characteristics, it can be considered an effective bioactive filler material. 

### 3.5. Hardness

**Figure 11:**
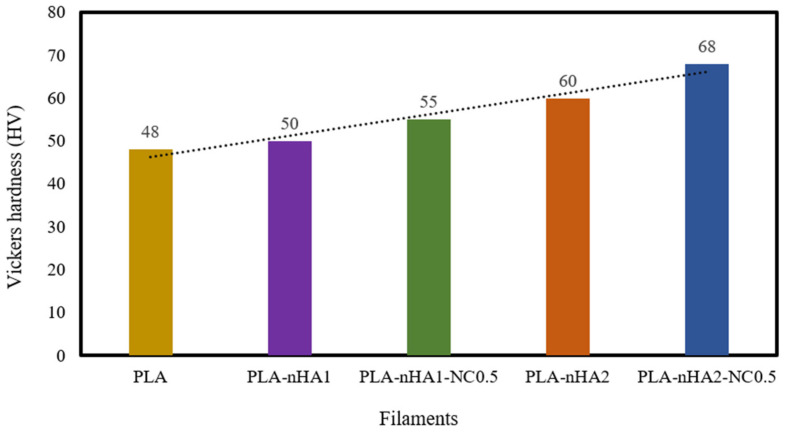
Vickers hardness of the neat PLA and its nanocomposite filaments.

The hardness of the neat PLA and the PLA nanocomposite filaments was examined. The results are shown in Table 5 and Figure 11. The reinforcement of the PLA matrix with nanoparticles, including nHA and NC, significantly improved the hardness of the neat PLA. Further, the addition of nHA particles at 1 and 2 wt% to the PLA matrix, respectively, increased the hardness value (HV) (50 and 60 HV) by 1.04 and 1.25 times, compared with the HV of the neat PLA (48 HV). The addition of the nHA–NC complex to the PLA matrix also enhanced its hardness. In fact, the HV of the PLA-nHA1-NC0.5 nanocomposite was higher than that of the neat PLA and PLA-nHA1, by 1.15 and 1.1 times, respectively. Meanwhile, the PLA-nHA2-NC0.5 nanocomposite had the highest HV at 68, higher than that of the neat PLA and PLA-nHA2 nanocomposite samples by 1.42 and 1.13 times. These results confirm that the addition of NC and nHA particles to the PLA matrix can improve the hardness of this polymer.

A study by Golan et al. [86] on the reinforcement of PLA with nHA and tungsten disulphide nanotubes showed that the HV of the PLA-nHA nanocomposite was higher than that of the neat PLA film by 1.4 times. Therefore, it was concluded that adding nHA particles to the PLA film increased the HV of the polymer. Another study by Arulmurugan et al. [87] investigated the nanomechanical characteristics of different ratios of montmorillonite clay-reinforced polyester composites. The addition of 5 wt% NC to the polymer matrix led to a 26.52% improvement in hardness compared with that of the neat polymer. Furthermore, the average Vickers HVs of cortical bone and cancellous bone are 0.396 and 0.345 GPa, respectively, as reported by Pramanik et al. [88]; the neat PLA and PLA nanocomposite samples in our study had higher HVs than cortical and cancellous bones. According to Wu et al. [89], bone HV typically ranges from 33.30 to 48.23 HV in different regions of the human body. From the perspective of hard tissue repairs such as bone or dental applications, the materials need to possess a high hardness ability that is required to replace the damaged tissue [90]. Therefore, the produced PLA nanocomposite filaments possess high hardness that can be utilised in hard tissue repairs.

### 3.6. Tensile Strength

**Figure 12:**
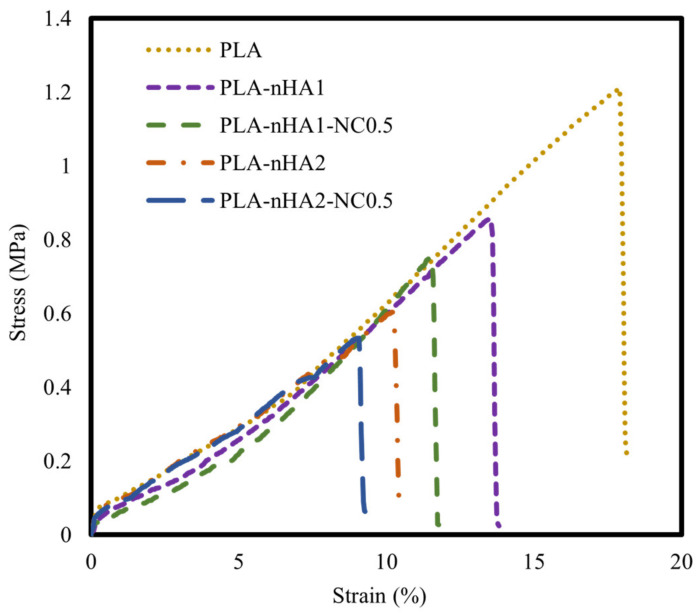
Tensile stress–strain curves of the neat PLA and different PLA nanocomposite filaments.

The tensile properties of the neat PLA and PLA nanocomposite filaments are illustrated using stress–strain curves in Figure 12 and mechanical characteristics in Table 6. The neat PLA had the highest tensile strength. Further, the tensile strength of the PLA matrix gradually decreased as the nanoparticle content increased. This phenomenon can be explained by the addition of inorganic additives, contributing to the decrease in PLA crystallinity. The decrease in the crystallinity led to a decrease in toughness and mechanical strength [91]. Furthermore, the poor interfacial interaction between the matrix and fillers displayed low load transferring, resulting in a decrease in mechanical strength. The decrease in the tensile strength of the nanocomposites could be attributed to the aggregation of nHA particles in the nanocomposites, considering that their surface energy is much higher than that of the PLA matrix [92]. Owing to the agglomeration of nHA, the PLA nanocomposites showed early failure and lower mechanical strength than the neat PLA. Han-Seung Ko et al. [93] reported that the tensile strength of PLA-nHA nanocomposites was lower than that of neat PLA owing to poor interfacial adhesion between the PLA matrix and nHA particles. Furthermore, Oliver-Ortega et al. [94] observed that the tensile strength of PLA was higher than that of PLA-NC nanocomposites.

### 3.7. Compressive Strength

**Figure 13:**
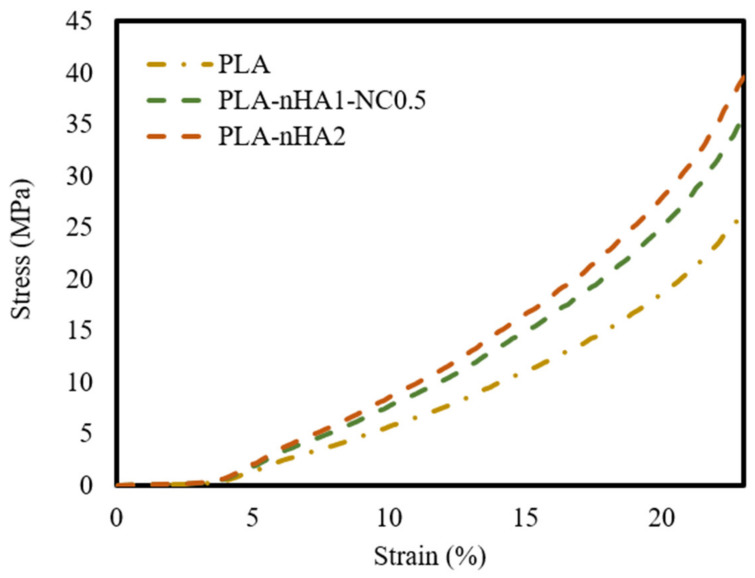
Stress–strain curves of the neat PLA and PLA nanocomposite 3D printed scaffold models.

The mechanical properties of the neat PLA and PLA nanocomposite 3D printed scaffold models were evaluated by compression testing. Additionally, the compressive elastic modulus, or Young’s modulus, as the slope of the linear part of the stress–strain curves, was calculated. The results are shown in Table 7. Figure 13 shows the stress–strain curves of the 3D scaffold model samples. The models demonstrated similar trends, with a linear zone in the range of 4–13% strain. Moreover, the deformation rate of PLA nanocomposite scaffold models was lower than that of the neat PLA scaffold model. Table 7 shows that the increase in Young’s modulus and compressive strength was proportional to the nanoparticles’ composition. In fact, Young’s modulus of the PLA increased by 1.45 times with the addition of 1% nHA and 0.5% NC and by 1.6 times with the addition of 2% nHA. The compressive strength of the PLA sample was the lowest at 27.22 MPa. Meanwhile, the compressive strength values of the PLA-nHA1-NC0.5 and PLA-nHA2 scaffold models were much higher, at 36.75 and 40.48 MPa, respectively. These results confirm that nHA and NC significantly enhanced the compressive properties of the PLA polymers.

It was found that the higher the nHA concentration, the higher the compressive strength and elastic modulus of the PLA-based scaffold [95,96]. From the perspective of tissue engineering applications, these outcomes show that the samples met the compressive strength criteria for trabecular bone, which ranges from 0.5 to 50 MPa under different types of stresses [97].

### 3.8. Water Absorption

**Figure 14:**
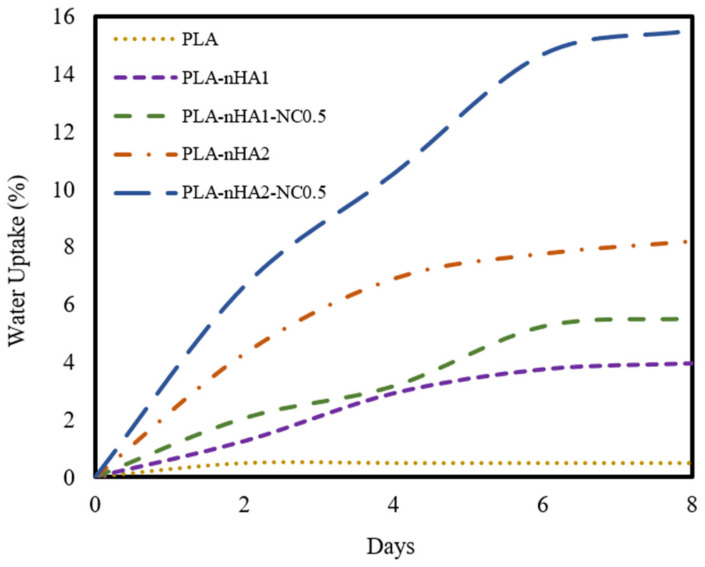
Water absorption of the neat PLA and PLA nanocomposite filament samples.

The water absorption behaviours of the neat PLA and PLA nanocomposite filaments are illustrated in Figure 14. The proportion of water uptake of the neat PLA was around 0.5%, and it reached equilibrium within 2 days. However, the various PLA nanocomposite filament samples showed rapid increases in water uptake in the first 4 days, with an insignificant surge after this period.

The water uptake percentage of the neat PLA was the lowest, while that of the PLA-nHA2-NC0.5 was the highest. The percentage of water absorption increased when the loading of nanoparticles increased. The weights of the neat PLA and PLA nanocomposite filament samples (PLA-nHA1, PLA-nHA1-NC0.5, PLA-nHA2, PLA-nHA2-NC0.5) increased by 0.5%, 4.0%, 5.5%, 8.5% and 15.5%, respectively, after 8 days of immersion in DI water. Therefore, the added nHA and NC in the nanocomposites played a key role in increasing the water absorption of the nanocomposite filaments. Water absorption is necessary for cell development and cell proliferation; hence, the high water absorption of the nanocomposite filaments indicates their potential as biocompatible materials [98].

### 3.9. Water Contact Angle

**Figure 15:**
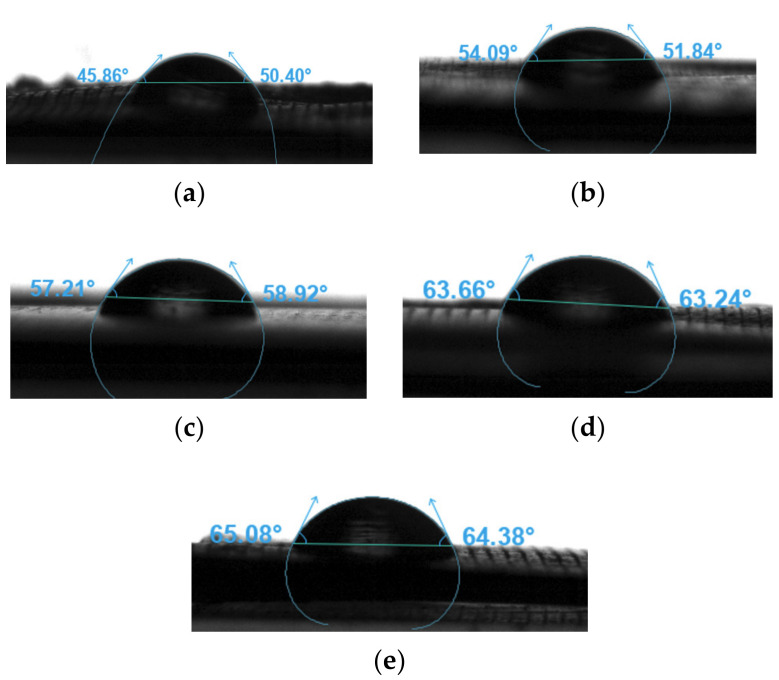
Sensile drop images of the filament samples (**a**) PLA, (**b**) PLA-nHA1, (**c**) PLA-nHA1-NC0.5, (**d**) PLA-nHA2, and (**e**) PLA-nHA2-NC0.5.

The water contact angles were measured to assess the wettability of the neat PLA and PLA nanocomposite filaments. Figure 15 presents the sessile drop images of the different samples, and the results are summarised in Table 8. The water contact angles of all samples were smaller than 90°. Therefore, these samples were hydrophilic. Moreover, the water contact angle increased as the concentration of the nanoparticles increased [99]. Table 4 shows that the PLA sample had the smallest contact angle at 48.13°, whereas the PLA-nHA2-NC0.5 sample had the largest contact angle at 64.73°. Although nHA is a hydrophilic biomaterial that contains hydroxyl groups, the addition of NC increased its hydrophobicity compared to that of neat PLA.

Wang et al. [100] reported that PLA-nHA nanocomposite membranes were more hydrophobic than neat PLA due to the addition of nHA. This result can be explained by the fact that the surface of the nanocomposite membranes was rough, which was disadvantageous for water attachment. Further, nHA and PLA are hydrophobic materials. Therefore, since water cannot penetrate the pores or voids of the rough surface, it rests on semi-solid and semi-air surfaces, thus increasing the contact angle [100]. The findings of the current study indicate that the surface hydrophobicity of the PNC increased with an increase in composite content.

### 3.10. Printability

**Figure 16:**
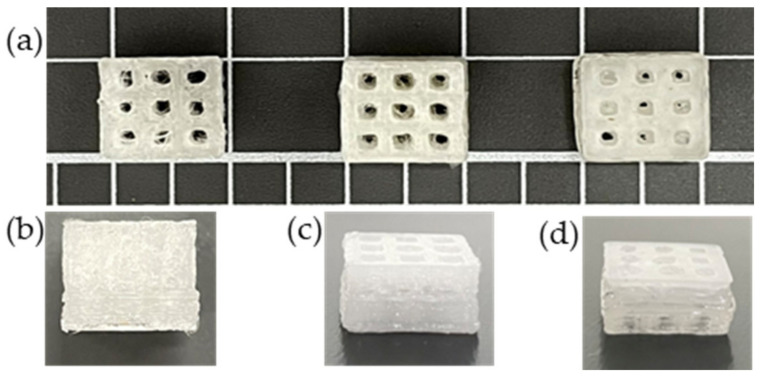
3D scaffold models were printed from the extruded PLA and PLA nanocomposite filaments: (**a**) front images of scaffold models; side images of (**b**) PLA, (**c**) PLA-nHA1-NC0.5, and (**d**) PLA-nHA2 scaffold models.

The 3D printed scaffold models were examined for strand printability and printing accuracy. Figure 16a–d show that the presence of nHA and NC particles insignificantly improved these printing features of the PLA polymer.

**Figure 17:**
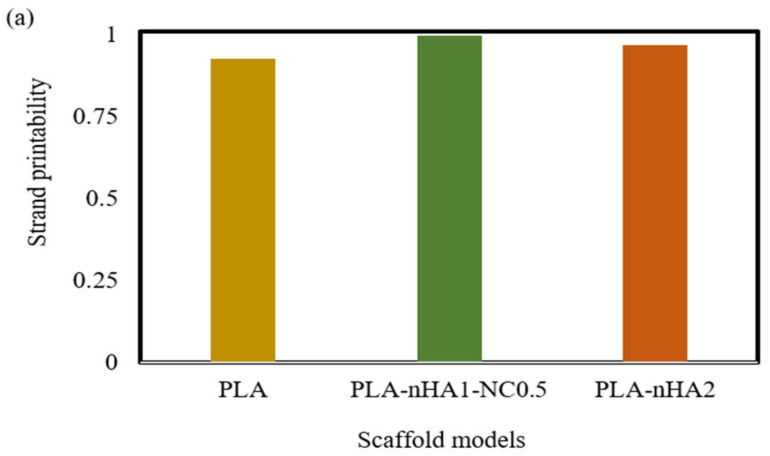

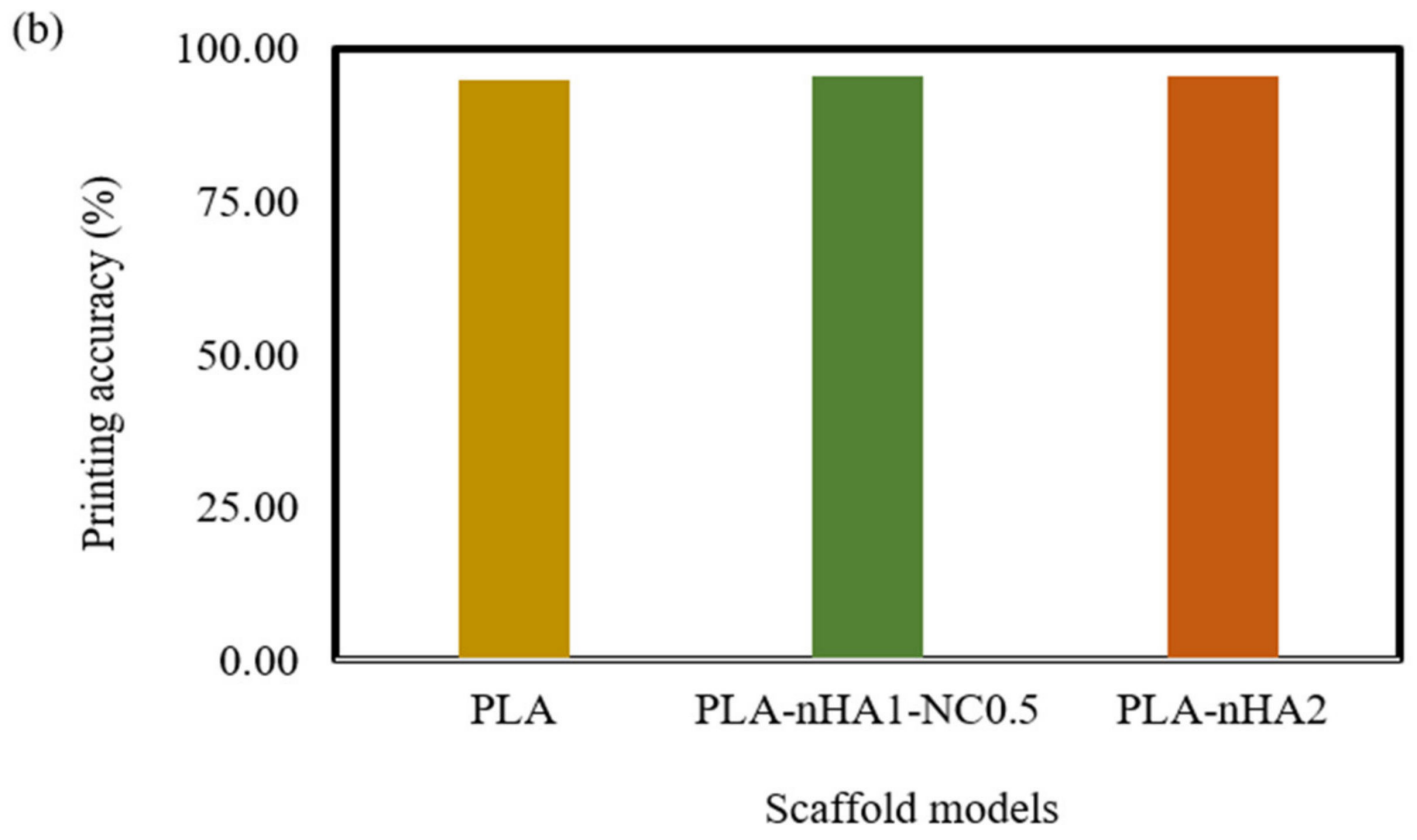
(**a**) Strand printability and (**b**) printing accuracy of different 3D printed scaffold models.

As mentioned in the methodology, strand printability is the ratio of the length of the printed strand to the length of the designed strand, so the ratio is deemed acceptable when it is close to 1. As illustrated in Figure 17a, the strand lengths of all the samples were smaller than that of the designed scaffold model, with a strand printability of <1. Therefore, the PLA-nHA1-NC0.5 scaffold model had the highest strand printability, at 0.996. Meanwhile, the strand printability values of the neat PLA and PLA-nHA2 scaffold models were lower, at 0.925 and 0.967, respectively.

However, Figure 17b confirms that the addition of nHA and NC particles to the compositions insignificantly enhanced the printing accuracy of PLA. The PLA nanocomposite scaffold models had a printing accuracy of around 95.6%, while the printing accuracy of the PLA scaffold model was about 95%. Figure 17b shows no considerable difference in the printing accuracies of the PLA nanocomposite printing, such as seen in the PLA-nHA1-NC0.5 (Figure 16c) and PLA-nHA2 scaffold models (Figure 16d). In comparison to PLA, PLA nanocomposites showed minimal improvement in printing accuracy.

## 4. Conclusions

The solvent casting method was used to prepare PLA reinforced with nHA and NC composites. The neat PLA and PLA nanocomposite filaments were fabricated using a melt extrusion procedure. The different characteristics of the extruded filaments were also investigated. First, the SEM images of obtained filaments revealed the presence of irregular-shaped nanoparticles on the fractured surface of PLA nanocomposite filaments in isolated and agglomerated forms. Second, the addition of nHA and NC to the PLA matrix contributed to the increase in the water absorption and water contact angle of the nanocomposites. Various mechanical properties of the acquired filaments were examined. The results demonstrated an increase in hardness but a decrease in tensile strength when nHA and NC particles were added to the PLA matrix. Furthermore, the thermal properties, including thermal stability and thermal behaviour, of the different filaments had improved in the presence of nHA and NC particles. Accordingly, different 3D scaffold models with grid-like structures (10 (L) × 10 (W) × 5 (T) mm^3^) were obtained by a 3D printer using the fused deposition modelling (FDM) technique. The outcomes confirmed the printability of the fabricated PLA nanocomposite filaments, with high printing accuracy (>95%). Further, reinforcement with nHA and NC did not play a role in enhancing the strand printability and printing accuracy of the PLA polymer. Additionally, the mechanical characteristics of the printed scaffold models were examined. We found that the presence of nHA and NC particles had significantly improved the compressive strength of the PLA polymer. Additionally, FTIR analysis confirmed the formation of nHA powder and the presence of various typical groups, including ${PO}_{4}^{3-}$, ${CO}_{3}^{2-}$ and ${OH}^{-}$.

## Figures and Tables

**Table 1:** Different heat treatment conditions applied to synthesise nHA powders.

Synthesised nHA Powders	Heat Treatment Conditions
nHA0	No sintering
nHA800	Sintering at 800 °C
nHA1000	Sintering at 1000 °C

**Table 2:** Different compositions of PLA nanocomposites.

Composition	PLA (wt%)	nHA (wt%)	NC (wt%)	Chloroform (mL)
PLA	100	0	0	10
PLA-nHA1	99	1	0	10
PLA-nHA1-NC0.5	98.5	1	0.5	10
PLA-nHA2	98	2	0	10
PLA-nHA2-NC0.5	97.5	2	0.5	10

**Table 3:** FilabotEX2 filament extrusion parameters.

Filaments	Nozzle Temperature (°C)	Speed of Printing (%)	Retraction Speed (%)	Fan Speed (%)
PLA	174 ± 1.5	50	5.5	10
PLA-nHA1	174 ± 1.5	50	5.0	10
PLA-nHA1-NC0.5	174 ± 1.5	50	5.0	10
PLA-nHA2	174 ± 1.5	50	5.0	10
PLA-nHA2-NC0.5	174 ± 1.5	50	5.0	10

**Table 4:** Glass transition, crystallisation and melting temperatures of the neat PLA and various PLA nanocomposites.

Sample	Cooling		Second Heating	
	T_g_ (°C)	T_c_ (°C)	T_g_ (°C)	T_m_ (°C)
PLA	55	N/A	67	174.5
PLA-nHA1	N/A	103	N/A	174
PLA-nHA1-NC0.5	N/A	102	N/A	174
PLA-nHA2	N/A	101	N/A	173.5
PLA-nHA2-NC0.5	N/A	96	N/A	173.5

**Table 5:** Vickers hardness of the neat PLA and PLA nanocomposite samples.

Samples	Vickers Hardness (HV)	Vickers Hardness (GPa)
PLA	48	0.470
PLA-nHA1	50	0.490
PLA-nHA1-NC0.5	55	0.539
PLA-nHA2	60	0.588
PLA-nHA2-NC0.5	68	0.666

**Table 6:** Mechanical characteristics of PLA-HA-NC composites.

Sample	Young’s Modulus (MPa)	Ultimate Tensile Strength (MPa)
PLA	16.04	1.20
PLA-nHA1	15.06	0.85
PLA-nHA1-NC0.5	16.45	0.75
PLA-nHA2	15.09	0.59
PLA-nHA2-NC0.5	16.48	0.54

**Table 7:** Young’s modulus and compressive strength of 3D printed scaffold models.

Scaffold Models	Young’s Modulus (MPa)	Compressive Strength (MPa)
PLA	1.66	27.22
PLA-nHA1-NC0.5	2.4	36.75
PLA-nHA2	2.66	40.48

**Table 8:** Water contact angle measurements of the neat PLA and PLA nanocomposite filaments.

Samples	Water Contact Angle (°)
PLA	48.13
PLA-nHA1	52.97
PLA-nHA1-NC0.5	58.01
PLA-nHA2	63.45
PLA-nHA2-NC0.5	64.73

## Data Availability

Not applicable.
